# Exercise intervention on the brain structure and function of patients with mild cognitive impairment: systematic review based on magnetic resonance imaging studies

**DOI:** 10.3389/fpsyt.2024.1464159

**Published:** 2024-12-02

**Authors:** Jing Xu, Jiangsheng Yu, Gai Li, Yanqiu Wang

**Affiliations:** Department of Physical Education and Sports, Central China Normal University, Wuhan, China

**Keywords:** exercise intervention, mild cognitive impairment, alzheimer’s disease, magnetic resonance imaging, systematic review

## Abstract

**Objective:**

This systematic review evaluates the impact of exercise intervention in MCI patients and discusses the potential neural mechanisms.

**Methods:**

A systematic search and screening of relevant literature was conducted in English and Chinese databases. Based on predefined keywords and criteria, 24 articles were assessed and analyzed.

**Results:**

Structurally, a significant increase was observed in the hippocampal and gray matter volumes of MCI patients following exercise intervention, with a trend of improvement in cortical thickness and white matter integrity. Functionally, after the exercise intervention, there were significant changes in the local spontaneous brain activity levels, cerebral blood flow, and functional connectivity during rest and memory encoding and retrieval tasks in MCI patients.

**Conclusion:**

Exercise may contribute to delaying neurodegenerative changes in brain structure and function in patients with MCI. However, the underlying neural mechanisms require further research.

**Systematic Review Registration:**

https://www.crd.york.ac.uk/PROSPERO/, identifier CRD42023482419.

## 1 Introduction

Mild cognitive impairment (MCI) represents a transitional state between normal aging and Alzheimer’s disease (AD), with approximately half of MCI patients progressing to AD within five years (1). Amidst the global aging, AD is a significant public health challenge, anticipated to affect over 150 million individuals by 2050 (2–5). Unlike AD, individuals with MCI are capable of self-care despite a noticeable decline in cognitive abilities (6). Given the irreversible progression of AD, it is an emerging priority to reduce the conversion rate from MCI to AD (7, 8).

Currently, clinical diagnosis of MCI patients typically involves a combination of neuropsychological assessments and neuroimaging examinations (9). Early neuropsychological assessments reveal declines in episodic memory, language, executive function, and processing speed across multiple cognitive domains in MCI patients (10–12). Non-invasive neuroimaging biomarkers further reveal neurodegenerative changes in the brain structure and function of MCI patients, closely associated with cognitive decline (13, 14). Among brain structures, the hippocampus plays a crucial role in the consolidation and retrieval of memories. It is a critical hub for higher executive functions, impulse control, cognitive flexibility, and decision-making within the brain network (15, 16). Compared to normal aging, MCI patients exhibit significant atrophy of the hippocampus, which disrupts neural circuit functions, leading to changes in the volume of other structures in the memory circuit and glucose metabolism in the hippocampus, resulting in a decline in cognitive abilities (17, 18). Meanwhile, MCI patients also show reduced cortical thickness and gray matter volume shrinkage related to cognitive decline (19, 20). The shrinkage in cortical thickness is an essential biomarker for identifying MCI patients (21), and the reduction in gray matter volume is associated with the progression of the disease in MCI patients (17). Furthermore, studies imaging the brain’s white matter *in vivo* in MCI patients have found that the integrity of white matter in multiple brain regions is compromised, and this damage may occur before detectable changes in brain volume (22). At the functional level, the functional connectivity of the brain in a resting state, by reflecting neuronal activity in the absence of goal-directed tasks and external inputs, helps to understand the interaction patterns of different brain networks (23). In the resting state of the brain, the default mode network (DMN) typically shows decreased activity during cognitive demand tasks and increased activity at rest (24). MCI patients show impaired functional integrity in major networks such as the DMN, and the extent of impairment correlates with disease progression (25), making functional connectivity in the resting state a diagnostic tool for MCI with medium to high reliability (26). Simultaneously, studies monitoring spontaneous neuronal activity in the brains of MCI patients have found abnormal activity in multiple brain regions, such as the frontal, temporal, and occipital lobes, which correlates with a decline in cognitive abilities (27–29). Additionally, a meta-analysis found that cerebral blood flow in ten brain regions, including the anterior cingulate cortex, is reduced in MCI patients, and the trend of decreased blood flow correlates with the severity of the disease (27).

Compared to the slow progression and limitations associated with drug consumption, exercise has shown potential as an economically viable, accessible, and safe intervention for maintaining, delaying, or improving cognitive function and brain health in both healthy older adults and MCI patients (30–32). In terms of cognitive function, meta-analyses indicate that exercise offers the greatest benefits for overall cognition, executive function, and memory in healthy older adults compared to younger populations (33), and that it can moderately improve cognitive function in MCI patients as well (34–36). In terms of brain health, initial studies have examined the effects of exercise on brain structure and function across MCI patients, AD patients, and healthy older adults, finding that exercise mainly impacts brain regions vulnerable to neurodegeneration, such as the frontal, temporal, and parietal lobes (37). Subsequent studies focused specifically on healthy older adults, indicating that exercise is associated with greater brain volume and enhanced task-related activity (38, 39). However, most studies have utilized mixed samples, lacking focused exploration of specific structural and functional changes in MCI patients, limiting understanding the neural mechanisms by which exercise improves brain health in MCI patients.

This systematic review focuses on the effects of exercise on brain structure and function in MCI patients. Given the potential of exercise to delay cognitive decline in MCI patients and its value as an adjunctive therapeutic strategy, this review exclusively includes interventional studies on exercise in MCI patients, concentrating on structural and functional changes in the brain. To ensure the causality of findings, only randomized controlled trials and non-randomized controlled trials were included, as these study designs provide higher inferential power. This study employed a modified scoring system by Pitkälä (40) to evaluate the methodological quality of the included studies, providing a reference for future research. We evaluated changes in hippocampal volume, cortical thickness, gray matter volume, and white matter integrity for the structural review. For the functional review, we focused on functional connectivity, cerebral blood flow, and local spontaneous brain activity following exercise. We found that exercise interventions may improve brain function, as observed through reduced neurodegenerative changes. We conclude this review by discussing potential future research directions and implications by which exercise promotes brain health in MCI patients.

## 2 Methods

This research protocol has been pre-registered on the PROSPERO platform, with the registration number CRD42023482419.

### 2.1 Search strategy

The study strictly followed the PRISMA (Preferred Reporting Items for Systematic Reviews and Meta-Analyses) guidelines to ensure the systematic and comprehensive nature of the literature search (41). Three sets of search keywords were established, connected internally with “OR” and between groups with “AND”, utilizing Boolean operators for combination. The search strategy for English databases is illustrated in **Table 1**. Two researchers independently used the search keywords to conduct full-text or subject searches in Chinese and English databases for literature published up to June 1, 2023. Chinese databases included CNKI (China National Knowledge Infrastructure) and Wanfang Data; English databases included PubMed, Web of Science, Embase, and Cochrane Library. The EndNote software was used for literature de-duplication, which involved comparing titles, authors, and publication years (42). The study also reviewed previously published review articles and reference lists of studies considered for inclusion.

**Table 1:** Search strategy for English databases.

Step	Search strategy
1	exercise OR resistance training OR sports OR tai ji OR yoga OR qigong OR walking OR jogging OR dance OR baduanjin OR physical activity
2	magnetic resonance imaging OR MRI OR functional magnetic resonance imaging OR fMRI OR diffusion tensor imaging OR DTI
3	cognitive dysfunction OR mild cognitive impairment OR MCI OR cognitive decline OR cognition disorders
4	1 AND 2 AND 3

### 2.2 Inclusion and exclusion criteria

To ensure the quality of the included literature, strict inclusion criteria were developed based on the PICOS principles of evidence-based medicine, detailed into five modules: Participants, Intervention, Comparison, Outcomes, and Study design (41).

Inclusion criteria: At least one group in the intervention or control group should be MCI patients. The intervention must include an exercise component. When the control group consists of MCI patients, the control measures include health education, receiving standard care, maintaining a regular lifestyle, or non-exercise controls. When the control group consisted of healthy subjects, they received the same exercise intervention as the intervention group and utilized MRI technology to assess variables of interest. Study types include randomized controlled trials (RCT) or non-randomized controlled trials (nRCT).

Exclusion Criteria: Studies that are duplicate publications; MCI caused by other physiological reasons, such as vascular MCI; Non-Chinese and non-English literature, studies with incomplete data (e.g., missing critical outcome data), conference abstracts, and studies that were not peer-reviewed; studies on acute exercise interventions.

### 2.3 Literature screening and data extraction

A three-level review process was adopted to ensure the rigor and accuracy of the literature screening. The primary review was conducted by two researchers who assessed the relevance of each study to the research topic by reading the titles and abstracts. The secondary review involved two researchers reading the full text to determine if it met the inclusion and exclusion criteria. In cases of conflict in the primary and secondary reviews, a consensus was reached through group discussion. The tertiary review involved two researchers extracting data from the included literature, with a third person checking to ensure the accuracy and completeness of the data extraction.

A standardized data extraction form was developed for this study, which included extracting the following information: first author’s surname, year of publication, sample size, age of study subjects, intervention and control measures, components of exercise intervention (duration per session, weekly frequency, total intervention period), MRI measurement techniques, and MRI results post-intervention.

### 2.4 Literature quality assessment

This study evaluated the methodological quality of the included references using a scoring system modified by Pitkälä et al. (40). This system integrates elements from the randomized intervention trial criteria developed by the Cochrane Library (43), the PEDro scale from the Physiotherapy Evidence Database (44), and standards set by the evidence-based medicine working group (45, 46), with the addition of an item assessing participant compliance, totaling 13 items. Each item scored one point, with a maximum score of 13. A literature quality score of ≥11 points was classified as high quality, 7-10 points as moderate quality, and <7 points as low quality. The specific content evaluated in each item was presented in **Table 2**.

**Table 2:** Criteria for methodological quality assessment of literature.

Item	Content
A	Adequate and acceptable description of the randomization method.
B	Clear definition of the study population.
C	Accurate description of inclusion and exclusion criteria.
D	Sufficient statistical power to detect differences between groups.
E	Clear and effective definition of measurement methods and outcome indicators.
F	Similarity between groups at baseline, or adjustments made to outcome indicators as necessary.
G	Description of participant dropouts (e.g., flow chart, reasons for dropout, and at which stage of the study), and their inclusion in the analysis.
H	Analysis based on intention to treat.
I	Comparison of changes in outcome variables between the two groups.
J	Outcome assessors are blinded to participant group allocation.
K	Detailed and replicable description of the experimental intervention.
L	Description of participant compliance.
M	Reporting of complications.

## 3 Results

### 3.1 Summary of studies

**Figure 1:**
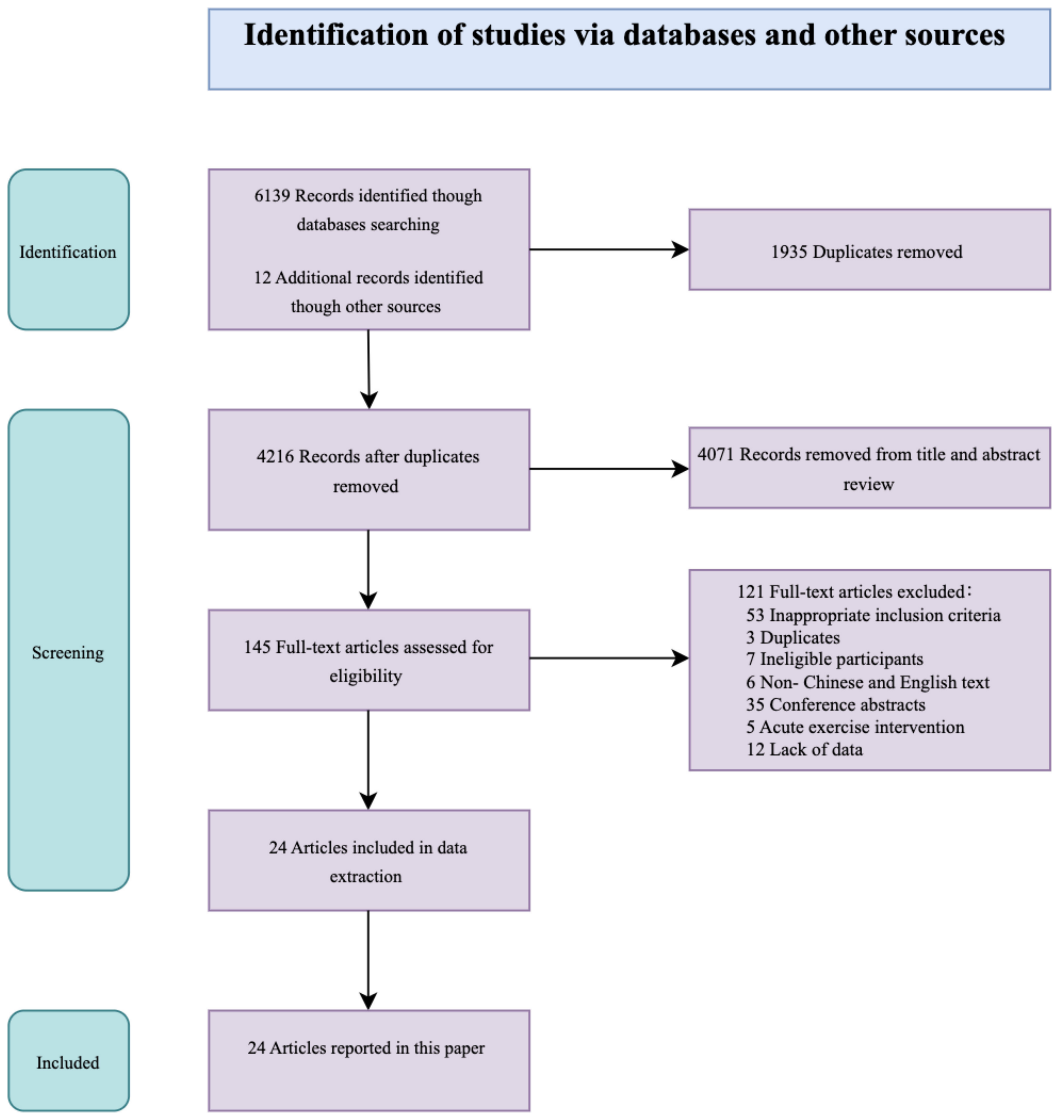
Flow diagram of literature selection.

This study identified 24 publications that met the inclusion and exclusion criteria, of which 17 were published within the last five years. The literature search flowchart is shown in **Figure 1**.

Classifying by study type, 7 were nRCT, and 17 were RCT. In nRCT studies, the intervention group consisted of MCI patients, while the control group comprised healthy older adults. In RCT studies, both the intervention and control groups comprised MCI patients.

The intervention groups used various methods, including aerobic exercise, resistance training, and mind-body exercises. The control groups engaged in health education, maintained their regular lifestyle, or performed stretching and toning exercises. The intervention period in each study was at least 12 weeks. The results regarding changes in brain structure and function covered various aspects, reflecting the comprehensiveness of the study findings. Structural change outcomes included hippocampal volume, cortical thickness, grey matter volume, and white matter integrity; functional change outcomes included task-based and resting-state brain FC, CBF, and ALFF. Detailed characteristics of the included literature are presented in **Table 3**.

**Table 3:** Detailed characteristics of the included literature.

Study ID	Design	Number of Participants	Age	Intervention Measures	Control Measures	Duration, Frequency, Period	Outcome Measures	Main MRI Results
Zhu et al., 2022 (47)	RCT	E=35MCI N=33MCI	71.51 ± 6.62 69.82 ± 7.74	Aerobic dance combined with health education	Health education	35 minutes, 3 times per week, 3 months	Hippocampal volume,	In the intervention group, right and total hippocampal volumes increased significantly compared to the control group; both groups experienced atrophy in the left hippocampus, but there was no significant intergroup difference.
Teixeira et al., 2018 (48)	nRCT	E=20MCI N=20MCI	69.4 ± 5.2 68.9 ± 5.5	Multicomponent exercise	Maintaining regular life	30 minutes, 3 times per week, 6 months	Hippocampal volume, Cortical thickness	The intervention group showed a significant increase in left and right hippocampal volumes, with no substantial changes in the control group. The intervention group exhibited an increasing trend in cortical thickness across various regions, while the control group showed a shrinking trend, but there were no significant differences between groups.
Tarumi et al., 2018 (49)	RCT	E=31MCI N=39MCI	64 ± 5.9 65.3 ± 6.6	Walking and other aerobic exercises	Stretching and toning	25-40 minutes, 3-5 times per week, 1 year	Hippocampal volume	There was no significant difference in hippocampal volume between the two groups; in amyloid-positive patients, hippocampal atrophy in the intervention group was significantly less compared to the control group. but this change was not significantly associated with changes in cognitive measures.
Broadhouse et al., 2020 (42)	RCT	E=27MCI E=22MCI E=24MCI N=27MCI	69.5 ± 6.6	Resistance training combined with computer cognitive training, resistance training, computer cognitive training	Sham stimulus control	90 minutes, 2-3 times per week, 26 weeks	Hippocampal volume	Compared to the control group, resistance exercise protected hippocampal subregions susceptible to AD-related atrophy (left subiculum, left CA1, dentate gyrus). and this plasticity was associated with long-term cognitive therapeutic benefits.
Reiter et al., 2015 (50)	nRCT	E=14MCI N=16HC	78.85 ± 7.75 75.87 ± 6.9	Treadmill walking	Treadmill walking	30 minutes, 4 times per week, 12 weeks (44 sessions)	Cortical thickness	Besides a significant decrease in cortical thickness in the right fusiform gyrus, cortical thickness remained stable in both groups; compared to the HC group, the MCI group exhibited more health-related changes in cortical thickness in the left insula and left superior temporal gyrus.
Suzuki et al., 2012 (51)	RCT	E=50MCI N=50MCI	74.8 ± 7.4 75.8 ± 6.1	Multicomponent training	Health education	90 minutes, 2 times per week, 6 months (40 sessions)	Cortical thickness	There was no significant difference in whole-brain cortical atrophy between the intervention and control groups; subgroup analysis showed that whole-brain cortical atrophy in the amnestic MCI intervention group was significantly less compared to the control group.
Tao et al., 2019 (52)	RCT	E=20MCI E=17MCI N=20MCI	66.17 ± 4.17 64.32 ± 2.6 65.97 ± 5.66	Baduanjin training combined with health education or brisk walking training combined with health education	Maintaining regular activities and receiving health education	60 minutes, 3 times per week, 24 weeks	GMV, ALFF values, resting-state fMRI FC	Compared to the control group, the Baduanjin group showed a significant increase in GMV in bilateral ACC, a considerable rise in ALFF values in bilateral ACC, and a substantial increase in resting-state FC between the hippocampus and the right angular gyrus. Notably, the increase in ALFF values in the ACC was associated with improvement in cognitive function.
Liu et al., 2021 (53)	RCT	E=20MCI E=17MCI N=20MCI	66.17 ± 4.17 64.32 ± 2.6 65.97 ± 5.66	Baduanjin training combined with health education or brisk walking training combined with health education	Maintaining regular activities and receiving health education	60 minutes, 3 times per week, 24 weeks	Resting-state fMRI FC, GMV	Compared to the other two groups, the Baduanjin group showed a significant increase in GMV in the right ACC; compared to the control group, Baduanjin significantly increased resting-state FC between the right LC and left VTA and the right insula and right amygdala. Notably, the increase in rs-FC between the right LC and right insula was associated with corresponding cognitive functions.
Kobe et al., 2015 (54)	RCT	E=13MCI N=9MCI	70 ± 7.2 70 ± 5.2	Omega-3 fatty acids combined with aerobic exercise and cognitive stimulation	Omega-3 fatty acids combined with stretching	45 minutes, 2 times per week, 6 months	GMV	The control group showed a decrease in GMV in frontal, parietal, and cingulate cortices, while the intervention group maintained or increased GMV in these areas, with significant differences between groups.
Suo et al., 2016 (55)	RCT	E=27MCI E=22MCI E=24MCI N=27MCI	70.1 ± 6.7	Resistance training combined with computer cognitive training, resistance training, computer cognitive training	Sham stimulus control	90 minutes, 2-3 times per week, 26 weeks	GMV	Resistance exercise significantly increased GMV in the PCC. and this change was associated with a significant improvement in overall cognitive function.
Zhang et al., 2020 (56)	RCT	E=16MCI N=16MCI	70.6 ± 6.2 69.1 ± 8.1	Aerobic dance training combined with routine care	Routine care	35 minutes, 3 times per week, 3 months	Brain white matter integrity	The intervention group showed an increase in FA values in bilateral cingulate gyrus, bilateral hippocampus, and bilateral superior longitudinal fasciculus compared to pre-intervention. In contrast, the control group showed a decrease, with no significant inter-group differences.
Tarumi et al., 2019 (57)	RCT	E=16MCI N=20MCI	67 ± 7 66 ± 7	Walking and other aerobic exercises	Stretching and toning	25-40 minutes, 3-5 times per week, 1 year	Brain white matter integrity	There were no significant differences in brain white matter integrity between the intervention and control groups; however, improvements in cardiorespiratory fitness in both groups were associated with slowing the degeneration of white matter integrity. Notably, changes in white matter integrity and cardiorespiratory fitness were not related to cognitive test scores.
Eyre et al., 2016 (58)	RCT	E=14MCI N=11MCI	67.1 ± 9.5 67.8 ± 9.7	Yoga	Cognitive training	60 minutes, once per week, 12 weeks	Resting-state fMRI FC	Improvements in verbal memory performance were significantly associated with increased FC between the DMN and medial frontal cortex, ACC, right middle frontal cortex, PCC, and left lateral occipital cortex.
Chirles et al., 2017 (59)	nRCT	E=16MCI N=16HC	79.6 ± 6.8 76.1 ± 7.2	Walking exercise	Walking exercise	50 minutes, 4 times per week, 12 weeks	Resting-state fMRI FC	The MCI group showed significant enhancement in FC of the DMN with frontal and parietal regions from pre to post-intervention.
Xia et al., 2019 (60)	RCT	E=23MCI E=23MCI N=23MCI	65.79 ± 4.35 64.88 ± 3.3 65.86 ± 5.28	Baduanjin training combined with health education or brisk walking training combined with health education	Maintaining regular activities and receiving health education	60 minutes, 3 times per week, 24 weeks	Resting-state fMRI FC	Compared to the other two groups, the Baduanjin group showed a significant decrease in FC between the DAN and right rolandic operculum, right middle temporal gyrus, right pre-cuneus, and right fusiform gyrus. however, these changes were not significantly associated with changes in attention test scores.
Won et al., 2021 (61)	nRCT	E=16MCI N=16HC	78.8 ± 7.6 75.3 ± 7.4	Treadmill walking	Treadmill walking	30 minutes, 4 times per week, 12 weeks (44 sessions)	Resting-state fMRI FC	The MCI group showed a significant increase in FC between the hippocampus and right PCC pre- and post-intervention, with significant bilateral increases in the hippocampus and bilateral PCC. Notably, Greater hippocampal FC with right PCC were associated with improved logical memory recognition performance.
Won et al., 2020 (62)	nRCT	E=17MCI N=18HC	79.5 ± 6.8 76.5 ± 7.2	Treadmill walking	Treadmill walking	30 minutes, 4 times per week, 12 weeks	Resting-state fMRI FC	There were no significant changes in FC between the three cerebellar seed points and other brain regions in the MCI group.
Smith et al., 2013 (63)	nRCT	E=17MCI N=18HC	78.7 ± 7.5 76.0 ± 7.3	Treadmill walking	Treadmill wang	30 minutes, 4 times per week, 12 weeks	Task-state fMRI FC	During a semantic memory retrieval task post-intervention, the MCI group showed a significant decrease in activation intensity in 11 brain regions, including the frontal, temporal, and parietal lobes.
Seligmamn et al., 2021 (64)	RCT	E=13MCI N=14MCI	70.84 ± 5.53 71.92 ± 6.40	Aerobic training (stationary cycling)	Maintaining low-intensity physical activity	40 minutes, 3 times per week, 16 weeks	Task-state fMRI FC	The intervention group showed a significant increase in activation in several brain regions during the memory encoding process, primarily in frontal areas, with an expansion of neural synchrony to higher-order cognitive areas at the temporo-parietal junction and frontal lobe during complex information processing tasks.
Qi et al., 2019 (65)	RCT	E=16MCI N=16MCI	70.6 ± 6.2 69.1 ± 8.1	Aerobic dance training combined with routine care	Routine care	35 minutes, 3 times per week, 3 months	Resting-state fMRI ALFF values	The intervention group showed a significant increase in ALFF values in bilateral frontotemporal lobes, anterior cingulate cortex, and parahippocampal and entorhinal cortices. but these changes was not significantly associated with changes in cognitive measures.
Li et al., 2021 (66)	RCT	E=48MCI E=51MCI N=53MCI	65.6 ± 5.6 65.5 ± 7.2 66.6 ± 7.1	Tai Chi combined with cognitive training, cognitive training	Providing health advice	60 minutes, 2 times per week, 12 or 24 months	Resting-state fMRI ALFF values	The intervention group showed a significant increase in ALFF values in bilateral medial temporal lobes, temporal poles, PCC, and insular cortices.
Tomato et al., 2021 (67)	RCT	E=22MCI N=30MCI	64.8 ± 6.4 66.1 ± 6.8	Aerobic training	Stretching	25-40 minutes, 3-5 times per week, 1 year	Total brain CBF	Post-intervention, aerobic training increased total CBF in amnestic MCI patients. but this change was not significantly associated with changes in cognitive measures.
Alfini et al., 2019 (68)	nRCT	E=15MCI N=17HC	80.5 ± 5.8 76.5 ± 7.2	Walking exercise	Walking exercise	30 minutes, 4 times per week, 12 weeks	CBF	At baseline, left insular CBF in MCI patients was significantly higher than in the HC group; post-intervention, inter-group differences disappeared; the intervention significantly reduced CBF in the ACC and right inferior frontal gyrus in the MCI group.
Thomas et al., 2020 (69)	RCT	E=15MCI N=15MCI	66.4 ± 6.6 66.1 ± 7.2	Aerobic exercise	Stretching and toning	25-40 minutes, 3-5 times per week, 1 year	CBF	Post-intervention, compared to the control group, the intervention group showed a significant decrease in CBF in the PCC and a significant increase in the ACC; CBF in the hippocampus significantly increased post-intervention in the intervention group.

RCT, Randomized Controlled Trial; nRCT, Non-Randomized Controlled Trial; MCI, Mild Cognitive Impairment; HC, Healthy Control; AD, Alzheimer’s Disease; min, minute; E, Intervention group; N, Control group; GMV, Gray Matter Volume; ALFF, Amplitude of Low Frequency Fluctuations; fMRI, Functional Magnetic Resonance Imaging; FC, Functional Connectivity; CBF, Cerebral Blood Flow; FA, Fractional Anisotropy; ACC, Anterior Cingulate Cortex; LC, Locus Coeruleus; VTA, Ventral Tegmental Area; PCC, Posterior Cingulate Cortex; DMN, Default Mode Network; DAN, Dorsal Attention Network.

### 3.2 Methodological quality

We assessed the methodological quality of the included literature. The average score of the 24 articles was 9.25 out of 13. Among them, 6 articles scored ≥11 points and were considered high-quality literature; 17 articles scored between 7 to 10 points, classified as medium quality; and 1 article scored less than 7 points and was considered low quality. Common missing points included lack of description and analysis of participant dropouts, not performing analysis based on intention-to-treat, not blinding outcome assessors, and lack of reporting on complications during the intervention. Detailed methodological quality scores can be seen in **Table 4**.

**Table 4:** “Literature quality assessment.

Study ID	A	B	C	D	E	F	G	H	I	J	K	L	M	Total Score
Zhu et al. (47)	1	1	1	1	1	1	1	0	1	1	1	1	1	12
Teixeira et al. (48)	0	1	1	0	1	1	0	0	1	0	1	1	0	7
Tarumi (A) et al. (49)	1	1	1	1	1	1	1	1	1	1	1	1	1	13
Broadhouse et al. (42)	1	1	1	1	1	1	0	0	1	0	1	1	0	9
Reiter et al. (50)	0	1	1	0	1	1	0	0	1	1	1	1	0	8
Suzuki et al. (51)	1	1	1	1	1	1	0	0	1	1	1	1	1	11
Tao et al. (52)	1	1	1	1	1	1	0	0	1	0	1	1	0	9
Liu et al. (53)	1	1	1	1	1	1	0	0	1	0	1	1	0	9
Kobe et al. (54)	1	1	1	1	1	1	0	0	1	0	1	1	0	9
Suo et al. (55)	1	1	0	1	1	0	0	1	1	1	1	0	0	8
Zhang et al. (56)	1	1	1	1	1	1	0	0	1	0	1	1	0	9
Tarumi(B) et al. (57)	1	1	1	1	1	1	0	0	1	1	1	1	1	11
Eyre et al. (58)	1	1	1	1	1	1	0	0	1	2	1	0	0	9
Chirles et al. (59)	0	1	1	0	1	1	0	0	1	3	1	1	0	8
Xia et al. (60)	1	1	0	1	1	1	0	0	1	4	0	0	0	6
Won et al. (61)	0	1	1	1	1	1	0	0	1	5	1	1	0	8
Won et al. (62)	0	1	1	1	1	1	0	0	1	6	1	1	0	8
Smith et al. (63)	0	1	1	0	1	1	0	0	1	7	1	1	0	8
Seligmamn et al. (64)	1	1	1	1	1	1	0	0	1	8	1	1	0	9
Qi et al. (65)	1	1	1	1	1	1	0	0	1	9	1	1	0	9
Li et al. (66)	1	1	1	1	1	1	1	1	1	10	1	1	1	13
Tomato et al. (67)	1	1	1	1	1	1	0	0	1	11	1	1	0	10
Alfini et al. (68)	0	1	1	1	1	1	0	0	1	12	1	1	0	8
Thomas et al. (69)	1	1	1	1	1	1	0	0	1	13	1	1	1	11

The table includes a scoring system with different criteria (represented by the letters A to M) for assessing the quality of each article. The details of the scoring system are provided in **Table 2**. The total score for each article is given in the last column.

### 3.3 The impact of exercise intervention on the brain structure in MCI patients

#### 3.3.1 Hippocampal volume

Overall, four studies investigated the impact of exercise interventions on hippocampal volume in MCI patients, and all found significant changes after the interventions. Two studies found that, following exercise intervention, hippocampal volume in MCI patients was significantly higher than in the control group. However, these studies reported inconsistent results regarding changes in the left hippocampus volume (47, 48). Among them, interventions that combined dance, walking, jogging, and ball sports showed a significant increase in both left and right hippocampal volumes in MCI patients (48). However, studies that used dance interventions alone found a significant increase only in the right hippocampal volume, while the left hippocampal volume experienced atrophy (47). One study did not directly show group differences in hippocampal volume after exercise intervention. Still, significantly less hippocampal atrophy was observed in amyloid-positive MCI patients in the intervention group compared to the control group (49). Additionally, the most severe hippocampal atrophy in MCI patients occurs in the CA1 and subiculum subfields; based on this, another study investigated the impact of exercise interventions on hippocampal volume in these regions and found that exercise intervention significantly reduced atrophy in hippocampal subregions susceptible to AD (42). These findings suggest that exercise intervention plays a positive role in lowering the risk of hippocampal atrophy. However, it should be noted that different studies have shown variability in the intervention outcomes within different subregions of the hippocampus.

#### 3.3.2 Cortical thickness

Overall, three studies focused on the impact of exercise interventions on cortical thickness in patients with MCI, but significant changes post-intervention were limited. Except for one study that reported a significant decrease in the cortical thickness of the right fusiform gyrus (50), none found substantial increases in cortical thickness after the intervention, nor were there significant differences between groups (48, 50, 51). This suggests that the positive effects of exercise interventions on cortical thickness in MCI patients are limited.

However, despite the lack of significant results, there is a trend toward an increase in cortical thickness among MCI patients, and improvements in cardiopulmonary function induced by exercise are positively correlated with increases in cortical thickness (48, 50). Particularly noteworthy is that in a subgroup analysis of amnestic MCI patients, one study found significantly less whole-brain cortical atrophy in the intervention group compared to the control group (51). These results suggest that exercise may have a specific protective effect on the cortical thickness of MCI patients, especially for those with amnestic MCI.

#### 3.3.3 Grey matter volume

Overall, four studies focused on the impact of exercise interventions on Gray Matter Volume (GMV) in patients with MCI, and all reported significant changes following the interventions. Three studies found that after exercise intervention, the GMV of the cingulate cortex in MCI patients was significantly higher than in the control group. However, these studies reported inconsistent results regarding specific parts of the cingulate cortex GMV (52, 53, 55). Among these, one study observed an increase in GMV in the bilateral anterior cingulate cortex (52), another noted a rise only in the right anterior cingulate cortex (53), and a third study reported an increase in the GMV of the posterior cingulate cortex (55). Additionally, another study combined exercise interventions with nutritional supplementation and cognitive stimulation, finding that after the intervention, the GMV in the posterior cingulate cortex, frontal lobe, and parietal cortex of the intervention group was significantly higher than that of the control group (54). These findings indicate that exercise intervention has a positive effect on increasing GMV in the cingulate cortex of MCI patients, and combined interventions may have a broader positive impact on multiple essential brain regions.

#### 3.3.4 White matter integrity

Two studies investigated the impact of exercise interventions on white matter integrity in patients with MCI, but neither found significant differences between groups after the intervention (56, 57). One study showed that Fractional Anisotropy (FA) tended to increase in specific brain regions, such as the hippocampus, cingulate gyrus, and superior longitudinal fasciculus, after three months of dance training in the intervention group (56). This trend reflects potential improvements in brain white matter integrity. Another study found that enhanced cardiopulmonary function after aerobic exercise was related to improved white matter integrity in MCI patients (57). These findings indicate that the effects of exercise interventions on white matter integrity in patients with MCI are limited.

Overall, exercise has a positive impact on the brain structure in MCI patients, especially in terms of hippocampal volume and GMV of the cingulate cortex. These significant changes emphasize the potential effects of exercise intervention in slowing or preventing neurodegenerative changes associated with MCI.

### 3.4 The impact of exercise intervention on the brain function in MCI patients

#### 3.4.1 Functional connectivity

Overall, seven studies focused on the changes in resting-state functional connectivity in patients with MCI following exercise interventions, each targeting different regions of interest. Additionally, two studies examined the changes in task-state functional connectivity in MCI patients. Among the seven studies focusing on resting-state functional connectivity, two studies focused on the default mode network (DMN) (58, 59). One study found increased connectivity between the DMN and the frontal lobe, cingulate gyrus, and occipital lobe after exercise in MCI patients (58). The other study focused on the posterior cingulate cortex/precuneus of the DMN and found increased connectivity between the posterior cingulate cortex/precuneus and the frontal and parietal regions (59). In addition, the study found that after exercise intervention, the functional connectivity between the dorsal attention network, which is negatively correlated with DMN activation, and the right rolandic operculum, right middle temporal gyrus, right precuneus, and right fusiform gyrus decreased (60). Two studies focusing on the hippocampus as the region of interest (52, 61), and one study focusing on the ventral tegmental area and locus coeruleus as regions of interest (53), all found increased functional connectivity in these regions after exercise intervention. Specifically, the studies on the hippocampus found increased connectivity between the hippocampus and the right angular gyrus, as well as between the hippocampus and the right posterior cingulate (52, 61). The study focusing on the ventral tegmental area and locus coeruleus found increased connectivity between the right locus coeruleus and the left ventral tegmental area with the right insula and right amygdala (53). However, a study with the cerebellum as ROI did not find any significant changes in FC following the intervention (62). Overall, these results suggest that exercise intervention leads to significant changes in FC in multiple brain networks and regions in MCI patients during resting state, except for the cerebellum.

Two studies investigating task-related brain FC used different task paradigms. In a study using a semantic memory retrieval task, it was found that activation in relevant brain regions of MCI patients significantly decreased after exercise intervention (63). Another study observed a significant increase in frontal lobe activity in MCI patients after intervention in a memory encoding task, and during complex information processing tasks, neural synchrony improved in higher-order cognitive regions, such as the temporo-parietal junction and frontal lobe (64). These results indicate that after exercise intervention, significant changes occurred in the brain activation of MCI patients during task performance.

#### 3.4.2 Amplitude of low frequency fluctuations and cerebral blood flow

Some studies investigated changes in more fundamental physiological and signaling characteristics. Three studies focusing on the amplitude of Low frequency fluctuations (ALFF) values found that after exercise intervention, ALFF values in certain brain regions of MCI patients significantly increased, indicating an enhancement in the level of local spontaneous brain activity in these regions (52, 65, 66). One study (52) found that after exercise intervention, compared to the control group, MCI patients had significantly increased ALFF values in the bilateral anterior cingulate cortex. This change in ALFF values was associated with higher scores on the Montreal Cognitive Assessment. In contrast, another study (65) did not find a relationship between increased ALFF values and cognitive test results. This study observed increased ALFF values not only in the anterior cingulate cortex, but also in the bilateral frontal and temporal lobes, entorhinal cortex, and hippocampal cortex. Additionally, one study (66) found that after exercise intervention, compared to before the intervention, MCI patients showed significant increases in ALFF values in the bilateral medial temporal lobes, temporal poles, posterior cingulate cortex, and insular cortex. Meanwhile, the control group showed decreases in ALFF values in these regions. The authors suggested that the control group developed more AD-related features.

Three studies focused on changes in CBF, all of which found significant changes before and after the intervention (67–69). One study focused on overall cerebral blood flow (67), while the other two focused on regional cerebral blood flow (68, 69). The study on overall cerebral blood flow found that, after exercise intervention, the overall cerebral blood flow in MCI patients increased, but this was not related to changes in cognitive ability (67). In contrast, the studies on regional cerebral blood flow found relationships between changes in blood flow and cognitive abilities (68, 69). One study found that, after exercise intervention, the cerebral blood flow in the left insula and anterior cingulate cortex of MCI patients decreased, which was associated with improvements in language fluency (68). However, another study found that the intervention led to increased cerebral blood flow in the anterior cingulate cortex, which was related to improvements in episodic memory (69). Additionally, increased hippocampal blood flow and decreased posterior cingulate cortex blood flow were observed (69). These results suggest that cerebral blood flow in MCI patients is susceptible to exercise interventions, and changes in regional cerebral blood flow may be related to improvements in cognitive function. However, different studies show varying trends in regional cerebral blood flow changes.

Overall, exercise had a broad and significant impact on the brain function of MCI patients. These impacts were reflected in the changes in FC of brain networks and in the changes in FC during task-specific performance. Furthermore, changes in local spontaneous brain activity levels and cerebral blood flow further emphasize the potential value of exercise intervention in improving and maintaining brain health.

### 3.5 Relationship between imaging and cognitive performances

Among the 24 studies included, 13 analyzed the relationships between imaging results and cognitive measures. Eight studies reported associations between changes in brain structure and function with cognitive measures in MCI patients following exercise interventions. Specifically, improvements in the hippocampus and cingulate cortex, both structurally and functionally, were linked to cognitive outcomes. For example, exercise-related plasticity in AD-susceptible hippocampal subregions contributed to long-term cognitive benefits (42); increased FC between the hippocampus and right PCC improved logical memory recognition (61), while higher ALFF values in the ACC and increased GMV in the PCC correlated with overall cognitive function gains (52, 55). Additionally, Changes in CBF in the ACC also related to improvements in verbal fluency and memory (68, 69). Furthermore, enhanced resting-state FC in specific brain regions also showed associations with cognitive outcomes; for instance, increased connectivity within the DMN between the medial frontal cortex, ACC, right middle frontal cortex, PCC, and left lateral occipital cortex was related to improvements in verbal memory performance (58). Increased FC between the right locus coeruleus and the right insula, as well as between the right locus coeruleus and the right ACC also showed associations with overall cognitive improvements (53).

In contrast, five studies found no significant association between brain changes and cognitive measures. For instance, three studies on amnestic MCI patients reported no correlation between reduced hippocampal atrophy, changes in white matter integrity, or increased global CBF with episodic memory or executive function (49, 57, 69). Additionally, changes in FC within the dorsal attention network did not correlate with attention test results (60), nor did changes in ALFF values in the bilateral frontotemporal lobes, ACC, parahippocampal cortex, or entorhinal cortex relate to general cognition, memory, or executive function (65).

## 4 Discussion

The objective of this review was to evaluate findings from MRI studies on the impact of exercise interventions on brain structure and function in patients with MCI. The results showed that, at the structural level, exercise intervention helps to slow hippocampal atrophy in MCI patients, increase gray matter volume in regions such as the cingulate cortex, and show a trend toward improvement in cortical thickness and white matter integrity. At the functional level, significant changes in functional connectivity were observed in multiple brain regions of MCI patients at rest after exercise intervention. These regions include the default mode network, dorsal attention network, hippocampus, locus coeruleus, and ventral tegmental area. Additionally, MCI patients exhibited significant changes in functional connectivity during memory encoding and retrieval tasks, as well as in local spontaneous brain activity levels and cerebral blood flow. Despite the heterogeneity in experimental design and methods across different studies, which led to varying experimental results, we still observed common improvement patterns, such as changes in hippocampal volume and gray matter volume in the cingulate cortex. The following sections will investigate the mechanisms underlying these changes, their clinical implications, and future research directions.

### 4.1 The impact of exercise intervention on the brain structure in MCI patients

#### 4.1.1 Hippocampal volume

The reviewed studies indicate that exercise led to significant changes in the hippocampal volume in MCI patients, manifested as either an increase in volume or a reduction in atrophy (42, 47–49). This is broadly consistent with the results of a previous meta-analysis, which included a demographic of elderly individuals encompassing healthy subject, MCI patients, AD patients, and people with diabetes, revealing that exercise interventions could have a positive effect on hippocampal volume, including in older populations vulnerable to hippocampal atrophy (70). The potential mechanisms of these changes may be related to exercise increasing the levels of neurotrophic factors, which are highly concentrated in the hippocampus and cerebral cortex (71). However, it is worth noting that in two randomized controlled trials studying the effects of exercise intervention on hippocampal volume in AD patients, no significant changes in hippocampal volume were found (72, 73). In summary, although exercise interventions have shown promise in increasing hippocampal volume and potentially mitigating atrophy across various populations, including those with MCI and healthy older adults, their effectiveness appears limited in AD patients. These findings underscore the complexity of AD pathology and the necessity for further research on the impact of exercise at different stages of cognitive decline.

Different exercise interventions have varying effects on the hippocampal volumes of the left and right hemispheres in MCI patients. For instance, multi-component interventions (combining outdoor walking, jogging, ball sports, and dance) can increase the volume of both hippocampi, while aerobic dance primarily affects the right hippocampus (47, 48). This variation might relate to the distinct roles of the hippocampi in memory processing: the right hippocampus is involved with visual-spatial memory, and the left hippocampus with verbal memory (47). The enhanced left hippocampal volume through diverse exercise scenarios may also improve patients’ episodic memory abilities. Furthermore, studies indicate that brain atrophy in MCI and AD patients is asymmetrical, with more pronounced atrophy on the left side (42). Due to limited sample sizes, this study did not perform a quantitative analysis. Future research should delve deeper into how different exercise interventions affect hippocampal volumetry in MCI patients, which is vital for understanding the specific impacts of exercise on brain structure and for formulating targeted intervention strategies.

#### 4.1.2 Cortical thickness

We found that the positive impact of exercise intervention on the cortical thickness of MCI patients was limited. The prominent areas of brain cortical atrophy in MCI patients include the hippocampus, entorhinal cortex, temporal lobe, pre-cuneus, posterior cingulate cortex, and the temporo-parietal junction (74, 75). However, no significant increases in cortical thickness were observed in these notably atrophied areas before and after the intervention, nor were there significant differences between groups (48, 50, 51). Research indicates that healthy elderly individuals and MCI patients share overlapping regions of cortical atrophy in their brains (76). Previous studies on exercise interventions in healthy older adults fail to demonstrate significant positive changes in cortical thickness (77, 78). These findings suggest that a single exercise intervention may not be sufficient to reverse cortical atrophy associated with aging and disease.

Despite the lack of significant changes, after exercise intervention, MCI patients showed a trend in increased cortical thickness and a positive correlation between improved cardiopulmonary function and cortical thickness (48, 50, 51). This suggests that changes in cortical thickness do not primarily reflect the protective effects of exercise on MCI patients. They are more likely dependent on alterations in cardiovascular responses. Furthermore, the duration of training may be a critical factor. The three studies included in our research had a total intervention time of no more than six months, yet significant intervention effects may not emerge until after 12 months (79).

Dual-task intervention models, such as combining exercise with cognitive tasks, showed a significant reduction in cortical atrophy in amnestic MCI patients compared to the control group (51). Previous dual-task intervention studies in community-dwelling older adults with cognitive decline (Not formally diagnosed with MCI) showed an increased cortical thickness in the temporal lobe compared to the control group after the intervention (80). These findings suggest that, compared to a single exercise intervention, a dual-task paradigm in MCI patients can be more effective in improving cognitive functions (81). Future research should explore combining exercise intervention with cognitive interventions as a holistic approach to potentially enhance the brain structure and cognitive ability of MCI patients.

#### 4.1.3 Grey matter volume

Our study explored the impact of exercise interventions on the gray matter volume (GMV) in the brains of MCI patients, focusing mainly on the hippocampus and cingulate cortex. We found that gray matter volume in the cingulate cortex of the intervention group was increased than that of the control group (52–55). A previous meta-analysis showed that compared to the control group, MCI patients experienced pathological loss of GMV in both hemispheres of the hippocampus (82). However, we found only two studies that examined the hippocampus as an ROI, and neither showed significant intergroup differences in GMV after exercise intervention (52, 55). This outcome is inconsistent with the results of prior studies, where exercise was found to increase the hippocampal GMV in healthy adults across various age groups (83). The differences observed might be associated with the types of exercise interventions used in the two studies included in this research: Baduanjin exercise and resistance training. Studies have shown that higher levels of cardiorespiratory fitness are generally correlated with increased gray matter volume in the hippocampal region (84). However, the improvements in cardiorespiratory function from Baduanjin and resistance training interventions may be limited in patients with MCI (85, 86). These findings underscore the need for further research into how different brain regions respond to exercise in the context of MCI and highlight the complexity of the brain’s response to physical activity.

Another study used a combined intervention (exercise, cognitive stimulation, and nutritional supplementation) to examine GMV changes in the frontal, temporal, and parietal lobes besides the cingulate cortex. GMV was significantly higher in the intervention group after the intervention for all regions except the temporal lobe, where the hippocampus is located (54). This result suggests that the GMV in the hippocampal region responds differently to combined intervention than other brain areas. It should be emphasized that changes in GMV are not physiologically specific and may represent a series of changes such as angiogenesis, gliogenesis, and neurogenesis (87, 88). As such, these results should be interpreted with caution. This finding poses new challenges for future research, namely how to accurately quantify the impact of exercise intervention on GMV in different brain regions in MCI patients and the role of these changes in MCI management.

#### 4.1.4 White matter integrity

We found two exercise intervention studies that suggest that exercise has limited positive effects on the white matter integrity in MCI patients, only showing positive changes after intervention without significant intergroup differences (56, 57). This outcome is consistent with previous studies focusing on older people, investigating the impact of exercise interventions on the structure of brain white matter, where no significant effect of exercise on white matter integrity was observed (89, 90). However, these studies used traditional detection indicators, such as FA and MD.

Research indicates that FA is susceptible to influences from multiple aspects of brain tissue microstructure, potentially rendering it unable to detect subtle, health-related changes in myelin or axons caused by exercise interventions (91). A recent study employed the ratio of standardized T1 and T2 weighted images (T1w/T2w) as a measure of the impact of exercise interventions on the white matter integrity of healthy older adults, finding a significant increase in the T1w/T2w ratio, which reflects an improvement in white matter integrity (92). Future research should consider using advanced microstructural measurement methods and higher-order imaging models to accurately assess the impact of exercise interventions on white matter integrity in patients with MCI (93), particularly in areas with complex fiber structures where traditional models fall short.

### 4.2 The impact of exercise intervention on the brain function of MCI patients

#### 4.2.1 Functional connectivity

Two studies focusing on the FC of the DMN during the resting state found that FC in the DMN of MCI patients was enhanced after exercise intervention (58, 59). This enhancement might reflect a compensatory response of the brain, where increased FC in the DMN indicates improved brain adaptability (94). Interestingly, the FC enhancement in the DMN of MCI patients after exercise intervention contrasts sharply with the situation in AD patients. In AD patients, functional connectivity in the DMN usually shows disruptions during clinical symptoms, with severe dementia leading to a loss of overall connectivity in the DMN (95). This comparison indicates that the effects of exercise on the FC of the DMN differ between MCI patients and AD patients.

The modulation of norepinephrine and dopamine is crucial in improving cognitive functions (53, 96). In the locus coeruleus and ventral tegmental area, the main brain regions for releasing these catecholamines, exercise intervention enhanced FC in these regions in MCI patients (53). However, not all brain regions exhibit significant changes in FC. Studies using the cerebellum and amygdala as seed points did not observe significant effects of exercise intervention on FC in MCI patients (62). As current studies are predominantly based on ROI local area analysis, future research exploring changes in resting-state FC after exercise intervention in MCI patients could consider employing voxel-based or whole-brain analysis methods to more comprehensively understand the impact of exercise intervention on the functional networks of the brain in MCI patients.

Two studies examined the impact of exercise intervention on brain functional connectivity of MCI patients during task performances (63, 64). The studies showed heterogeneous results. This difference primarily manifests in the direction of changes in the activation of task-related brain regions. One study examined semantic memory retrieval tasks and found decreased activation in relevant brain regions (frontal lobe, temporal lobe, and parietal lobe). The other study examined memory encoding tasks and observed increased activation in related areas(inferior frontal gyrus, middle frontal gyrus, precentral gyrus, and lingual gyrus) (63, 64). These different activation patterns may reflect successful compensatory responses or increased neural processing efficiency (63). Regardless of the direction of activation changes, these changes can be interpreted as a result of the intervention.

One study found that functional changes induced by specific exercise interventions showed similarities across different task paradigms (64). This study tested brain activation during associative memory encoding and complex information processing tasks between the aerobic training group and the balance and stretching control group. For instance, aerobic training intervention caused an increase in brain activation in the frontal lobe regions in both task paradigms. In contrast, the coordination training control group experienced a decrease in brain activation in the posterior parietal cortex (64). This finding aligns with results from studies on normal aging individuals (97, 98). Specific types of exercise interventions can induce distinct changes in brain activation across different task paradigms. These results underscore the importance of further investigating how various exercise interventions specifically impact brain function, pointing toward a promising direction for future research in cognitive health.

#### 4.2.2 Amplitude of low frequency fluctuations and cerebral blood flow

Three studies focused on the impact of exercise on cerebral blood flow (CBF) in MCI patients and observed significant intervention effects (67–69). One study indicated that exercise intervention led to an overall increase in CBF in MCI patients, possibly due to a reduction in central arterial stiffness (67). This finding is encouraging since vascular risk factors in MCI patients, such as decreased blood flow and blood flow velocity, are associated with the degree of cognitive impairment (99). The other two studies focused on changes in local CBF, particularly in the anterior cingulate cortex, and showed contrasting results (68, 69). One study found that the intervention led to a significant increase in CBF in the anterior cingulate cortex (69). In contrast, the other study observed a significant decrease in CBF in the anterior cingulate cortex following the intervention (68). Future studies should interpret the intervention effects on CBF in a clinical context and explore the potential relationship between changes in CBF and amyloid-beta, a core pathology.

Three studies in this review found a significant increase in ALFF in MCI patients compared to the control group after the intervention (52, 65, 66). Although the corresponding brain areas varied, this substantial change in ALFF manifests the improvement in local brain area spontaneous neural activity in MCI patients due to exercise intervention (52, 65, 66). However, any changes in coupled or baseline cerebral blood flow, cerebral blood volume, or oxygen metabolism can lead to different changes in the BOLD signal (100). The three studies included did not measure these related variables, so the ALFF changes should be interpreted cautiously.

### 4.3 Relationship between imaging and cognitive performances

Among the 13 studies reporting both imaging and cognitive outcomes, 8 studies (42, 52, 53, 55, 58, 61, 68, 69) demonstrated associations between exercise-induced changes in brain structure and function with cognitive improvements in MCI patients, while the remaining 5 studies (49, 57, 60, 65, 69) found no such relationship. This suggests that changes in imaging and cognitive measures may not follow a linear correlation, as one may reach a plateau at a certain stage while the other continues to evolve. Notably, among the 8 studies showing associations, 6 reported a link between changes in the hippocampus and cingulate cortex and cognitive improvement, underscoring the importance of these regions in mitigating cognitive decline in MCI patients. This finding aligns with studies on healthy older adults, where cognitive function declines alongside hippocampal shrinkage during normal aging (101). Furthermore, functional connectivity and cerebral blood flow in the posterior cingulate are associated with cognitive performance (102, 103), and gray matter volume in the anterior cingulate cortex may serve as a neural reserve, playing a protective role against cognitive decline (104).

### 4.4 Limitations and prospects

Firstly, some of the included studies had small sample sizes, and such small-sample studies might affect the representativeness and reliability of the results. Future research needs a larger sample size and multicenter intervention experiments to verify the related results. Secondly, due to the heterogeneity and diversity of outcome indicators in the included studies, this study was unable to perform a meta-analysis at the level of brain structure nor an assessment of activation consistency based on brain activation point coordinates at the functional level. Future review studies should consider conducting quantitative meta-analyses to draw more definitive conclusions. Third, this study only discusses the overall impact of exercise on MCI patients, but different types of exercise may induce different physiological mechanisms (105). There is a lack of sufficient evidence to assess these interventional differences. Future research should refine the elements of exercise intervention (e.g., type, cycle, frequency, duration, intensity, etc.) and carry out comparative studies to provide a scientific basis for developing exercise prescriptions for MCI patients. Lastly, this study discusses the impact of chronic exercise intervention on the brain structure and function of MCI patients. Future research should explore the relationship between acute and chronic exercise in brain changes in MCI patients, promoting an understanding of the neurophysiological mechanisms behind exercise-induced brain health changes in MCI patients.

## 5 Conclusion

Overall, exercise intervention shows notable structural and functional effects on the brain in MCI patients. Structurally, these positive effects are primarily manifested in significant changes in hippocampal and grey matter volume and observed increases in cortical thickness and white matter integrity. Functionally, considerable changes are mainly evident in brain functional connectivity, local spontaneous brain activity levels, and cerebral blood flow. Although these findings are positive, the specific effects of different exercise intervention programs on the brain structure and function of MCI patients remain unclear. Further research is needed to understand the underlying neural mechanisms fully. Understanding these mechanisms is crucial for better interpreting the impact of exercise interventions and developing more effective treatment strategies that may delay cognitive decline.

## Data Availability

The original contributions presented in the study are included in the article/supplementary material. Further inquiries can be directed to the corresponding author.
